# Evaluation of the response to treatment of solid tumours – a consensus statement of the International Cancer Imaging Society

**DOI:** 10.1038/sj.bjc.6601843

**Published:** 2004-05-11

**Authors:** J E Husband, L H Schwartz, J Spencer, L Ollivier, D M King, R Johnson, R Reznek

**Affiliations:** 1 Academic Department of Diagnostic Radiology, Royal Marsden Hospital, Downs Road, Sutton, Surrey SM2 5PT, UK; 2 Magnetic Resonance Imaging (MRI), Memorial Sloan-Kettering Cancer Center, 1275 York Avenue, Room C-276, New York, NY 10021, USA; 3 Department of Clinical Radiology, Lincoln Wing, St James' University Hospital, Beckett Street, LEEDS LS9 7TF, UK; 4 Department of Radiology, Institut Curie, 26 Rue D'Ulm, Paris 75005, France; 5 The Royal Marsden NHS Trust, Fulham Road, London SW3 6JJ, UK; 6 Department of Radiology, Christie Hospital NHS Trust, Wilmslow Road, Withington, Manchester M20 4BX, UK; 7 Academic Department of Diagnostic Radiology, St Bartholomew's Hospital, Dominion House, 59 Bartholomew Close, London EC1A 7ED, UK

**Keywords:** response, solid tumours, imaging, RECIST, ICIS

## Abstract

New guidelines to evaluate the response to treatment in solid tumors using imaging techniques have major limitations and important implications for radiological workload. This consensus statement from the International Cancer Imaging Society (ICIS) reviews the RECIST criteria and addresses several challenges regarding tumour measurement. Recommendations are made regarding tumour measurement and other issues are raised. The growing need to introduce a multimodality approach to monitoring response is recognized. ICIS welcomes a dialogue with the authors of RECIST to address issues raised in this review.

New guidelines to evaluate the response to treatment in solid tumours were published in the Journal of the National Cancer Institute in February 2000 (Therasse *et al*, 2000). These new criteria were developed by a task force set up by the European Organisation for Research and Treatment of Cancer (EORTC), the National Cancer Institute (NCI) of the United States and the National Cancer Institute of Canada Clinical Trials Group in an attempt to address areas of conflict and inconsistency between different methods of assessing response used in clinical research throughout the world. It was hoped that these new criteria and guidelines for their use would provide a standardised practical approach, which could be adopted uniformly on a wide scale. In defining the new Response Criteria In Solid Tumours (RECIST), the group have recognised the pivotal role of imaging in response assessment. They have acknowledged the need for continuous appraisal of rapidly advancing imaging technology and are aware of the need to develop functional imaging to provide surrogate end points for ‘novel’ therapies. We applaud the reappraisal of guidelines for therapy response assessment, especially in the light of the rapid advances in medical imaging technology and oncologic therapies. We are also grateful for the attempt to standardise techniques in imaging included in RECIST; however, these new criteria and guidelines have major limitations as well as important implications for diagnostic radiological workload. In this communication, we address our concerns regarding the use of the RECIST criteria.

The International Cancer Imaging Society (ICIS) was founded in 1999 in order to promote education in cancer imaging and its role in the multidisciplinary management of malignant disease. This rapidly growing society currently has over 200 members worldwide and holds an annual scientific meeting and postgraduate teaching course. The vast majority of the associate members are radiologists who have an interest in cancer radiology and the 38 full members are all specialist oncological radiologists who are international leaders in the field.

At the second annual course of ICIS, which was held in London in October 2001, a symposium entitled ‘Monitoring Response to Treatment’ provided the forum for a discussion of the difficulties encountered in monitoring tumour response with imaging and, in particular, the practical challenges of adopting the new RECIST criteria. The discussion stimulated this review. We applaud the standardisation of response assessment criteria and in particular numerous features of RECIST, which address specific imaging guidelines in clinical trials and considerations of other modalities and techniques for response assessment. Many of the general problems of monitoring tumour response with imaging have been addressed in the literature previously (Thiesse *et al*, 1997). However, we would like to share our views regarding the strengths and weaknesses of using the RECIST criteria, and what other issues should potentially be added to a response criterion with a wider audience in the hope that we may be able to open discussion between ICIS and the Task Force of the EORTC, the NCI and the NCI (Canada). Our ultimate objective is to offer constructive suggestions on the guidance for implementation of the RECIST criteria.

- We recognise three important purposes for evaluation of treatment response for reporting the outcome of clinical trials, that is, tumour response as a prospective end point in early clinical trials, tumour response as a prospective end point in more definitive clinical trials designed to provide an estimate of benefit for a specific cohort of patients and tumour response as a guide for the clinician and patient or study subject in decisions about continuation of current therapy. We also acknowledge the need to assess tumour response in daily clinical practice. Although the protocols for imaging to assess response for clinical trials are likely to differ considerably from those required to assess individual clinical response in routine practice, the key to accurate, reproducible assessment, for both types of practice, is the involvement of a radiologist experienced in oncological imaging. The assessment of response not only requires an approximate estimate of tumour size, or more precise measurement, but also requires an in-depth understanding of the complications of cancer therapies and a detailed knowledge of the disease-specific patterns of tumour recurrence. The difference between imaging in clinical care and imaging for clinical trials is subtle, especially for the non-radiologist, but of paramount importance to obtain results that are reproducible and consistent across centres. In some manner, the difference in radiologic assessment is similar to differences in practice for the chemotherapist practising in clinical care versus enrolling and caring for a patient on a clinical trial.
- The new RECIST criteria do not allow for the comparison of historical trials using WHO and current trials using RECIST. The data from the eight clinical trials used in the RECIST documentation were not referenced in the published criteria and guidelines (Therasse *et al*, 2000). It is uncertain what modality was used in these trials, what methodology was used to calculate the measurements and how many tumours or metastases were actually measured. Subsequent data have demonstrated some concordance between RECIST and WHO for *best overall response* but limited or no concordance between RECIST and WHO for *time to progression* (Schwartz *et al*, 2001; Warren *et al*, 2001; Prasad *et al*, 2002; Wang *et al*, 2003). This criterion is increasingly being used as a surrogate end point and the lack of correlation between RECIST and WHO is of concern. Thus there is potential for inappropriate application of noncomparable standards, which could potentially result in imaging being viewed as a poor method for quantitatively judging response. Furthermore, new drugs and therapies may not be approved for clinical use or may not reach the stage of full evaluation because of the application of differing standards and the comparison to historical controls and clinical trials. However, when appropriately applied, imaging represents one of the best methods available for objective and quantifiable response assessment.

The RECIST criteria aim to replace bidimensional assessment of one or more target lesions with summation of unidimensional assessments of many lesions: up to five in a single organ; up to 10 in total. These measurements aim simply to reflect categories of treatment response as defined in the WHO criteria (Miller *et al*, 1981). The application of serial arithmetic assessment of the RECIST criteria corresponds well with those of WHO within the category of *partial response*. However, comparison of the unidimensional method (RECIST) and the bidimensional method (WHO) is discrepant for *progressive disease*. The RECIST criterion of 20% increase in length to indicate progressive disease (PD) does not equate to the 25% increase as judged by the WHO criteria. Indeed a 20% increase in a unidimensional measurement using the RECIST criterion equates to a 44% increase in bidimensional measurement (Sohaib *et al*, 2000). This discrepancy is partially discussed within the new guidelines, as a concern related to the difficulty of measuring lesions that increase by 12% unidimensionally (mathematically corresponding to the original bidimensional WHO 25%). However, the difficulty or impact of the change in response assessment for disease progression was not proven or justified in the guidelines. Clearly, such a discrepancy will arguably result in longer periods of progression-free disease and specifically in changes in time to progression between new trials using the RECIST criteria and those using WHO criteria (Warren *et al*, 2001; Schwartz *et al*, 2003a).

- There are several challenges regarding tumour measurements.
  - The RECIST criteria of adoption of a single measurement (long axis) state that measurement is to be made only in the axial plane for CT. With MR and multislice CT, longitudinal or oblique measurements as well as axial measurements can now be made readily. Three-dimensional (3D) assessment and measurement of solid tumours is now available in many institutions. Its widespread use will clearly depend upon penetration of the acquisition devices needed for these data as well as the advanced computer workstations needed to perform accurate 3D quantification. Three-dimensional measurement is not addressed within the new criteria but may be important in the future, particularly with the advent of highly sophisticated volumetric scanning techniques (including multichannel CT and volumetric MRI) capable of acquiring isotropic imaging data. The use of 3D volume measurement has already been substantiated in several studies (Eggli *et al*, 1995; Johnson *et al*, 1995; Sohaib *et al*, 2000; Sorenson *et al*, 2001).
  - Lymph node involvement by tumour is different from other common soft tissue tumour sites. It is well recognised, especially in the radiology literature, that lymph nodes should be measured in the short axis dimension as this is the best predictor of the presence of metastatic disease (Kim *et al*, 1994) (Figure 1

**Figure 1:**
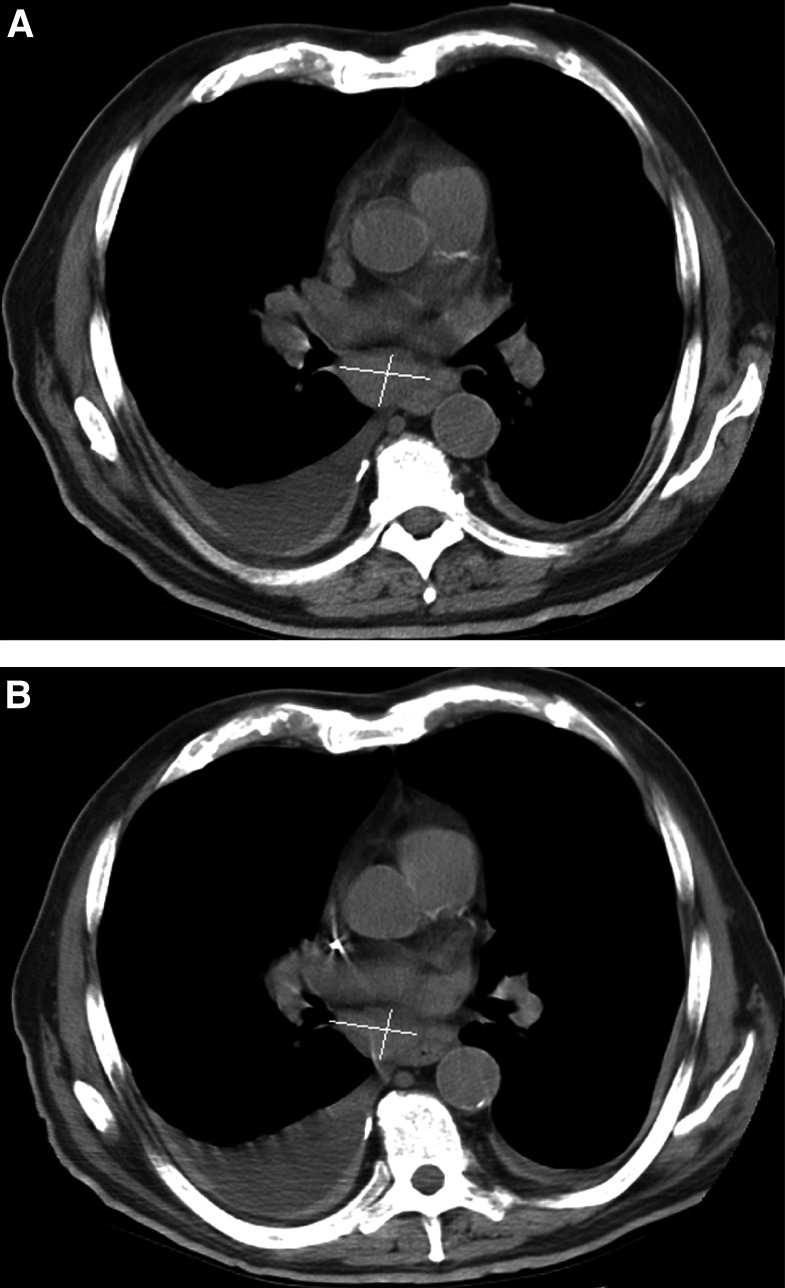
Patient with non-small cell lung cancer on chemotherapy. Evidence of enlarged subcarinal lymph node measuring 3.4 × 2.9 cm at baseline (**A**), which has decreased in size to 3.4 × 2.0 cm at subsequent follow-up (**B**).

). Furthermore, in complete remission after treatment, lymph nodes return to normal size yet the nodes may remain visible. However, *complete remission* cannot be designated to such nodes because the normal size of the nodes on an individual basis is not known. Specialised criteria have already been created to address this issue in non-Hodgkin's lymphomas (Cheson *et al*, 1999). Similar difficulties apply to designating complete remission in other structures, for example, the adrenal glands, the vaginal vault, bladder wall, etc.
  - Most tumours within the body (except lung metastases) grow irregularly and also regress in an irregular manner. It is true, and has been well documented that at baseline or any one time point, there is good correlation between the maximal tumour diameter, bidimensional measurement and tumour volume. However, tumours do not necessarily grow or shrink in a rounded fashion. In fact, lymph nodes frequently grow to a greater extent in the short axis (Jeong *et al*, 2003). Therefore, measurement of the longest diameter on sequential scans may not represent the true response.
  - Issues surrounding cystic tumours are problematic. The RECIST states that cystic lesions should be regarded as nonmeasurable. Many lesions have cystic/necrotic centres that may change on treatment and indeed metastases in the liver may be cystic *de novo* (e.g. non-seminomatous germ cell tumours, ovarian cancer). While it is recognised that certain ‘cystic tumours’ increase in size on effective treatment (e.g. non-seminomatous germ cell tumour metastases; Husband *et al*, 1982), the statement that all cystic lesions should be regarded as nonmeasurable is, in our view, too wide. Although tumours with cystic/necrotic centres should be avoided as targets if possible, tumours with cystic/necrotic centres may also be included in the assessment but a comment should be made documenting changes in tumour composition. In those tumour types where the phenomenon of ‘cystic enlargement’ is well recognised, for example, non-seminomatous germ cell tumours, appropriate note should be made. The criteria completely ignore other changes in lesion consistency such as calcification, necrosis or haemorrhage. These phenomena, which may develop only on treatment, may influence changes in tumour size. While such phenomena have no clear prognostic implications (Hale *et al*, 1998), it is only by documentation of such findings that progress in assessing response with morphological assessment will be made.
  - Another potential pitfall in simple tumour measurements in response assessment is the issue of lesion calcification. Calcification in response assessment may be seen in metastases to the lymph nodes, liver or peritoneum. Response assessment is not well assessed by simple measurement in these cases (Figure 2

**Figure 2:**
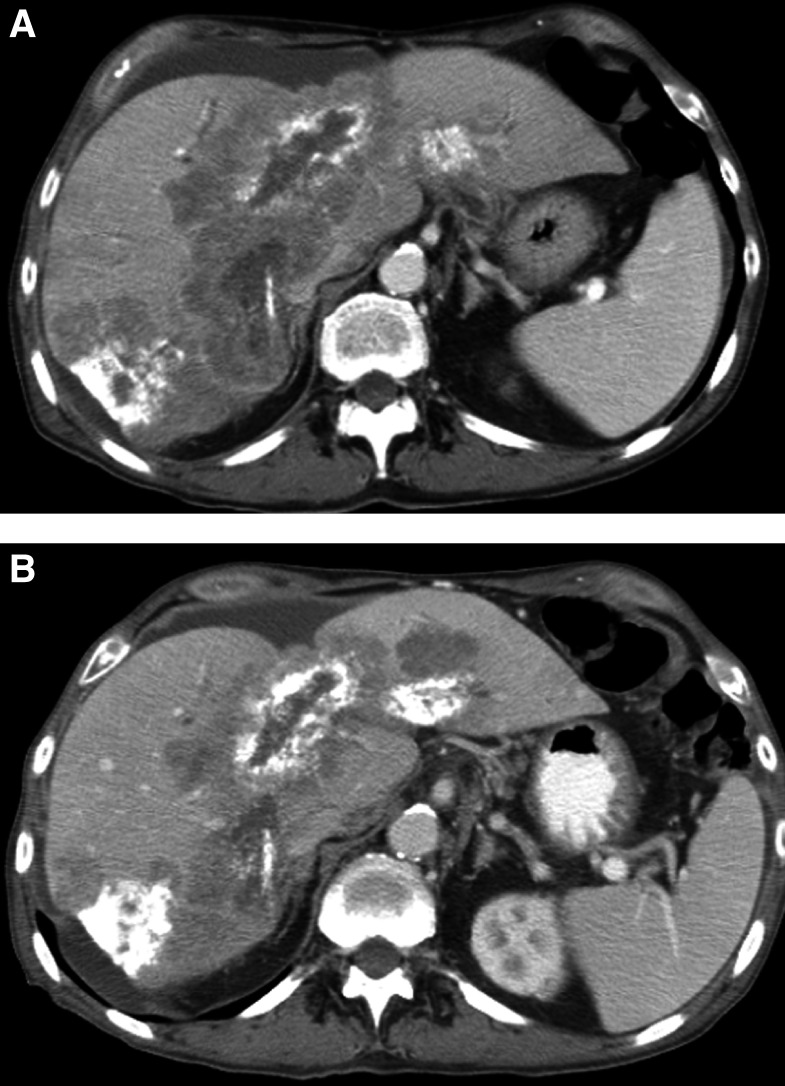
Partially calcified liver metastases from colorectal cancer imaged at baseline (**A**) and at a 6-week interval follow-up (**B**). The soft tissue masses surrounding the calcification have decreased in size – the overall mass including the calcification has either stayed stable or increased.

).
  - Other types of lesions are also difficult to measure unidimensionally or for that matter bidimensionally. For instance, en-plaque lesions frequently diminish in the short axis plane rather than in their long axis. Furthermore, the measurement of en-plaque lesion length can be particularly difficult as the plane of imaging is frequently in the same plane as the long axis of tumour growth.
  - For the first time, the RECIST criteria have provided imaging guidelines for both spiral and conventional CT, which is commendable and a great achievement, as it has standardised imaging protocols across sites performing these studies. The rapid advancement of imaging technology over the last few years has, however, rendered the protocols detailed in Appendix I outdated in many radiology departments undertaking clinical trials. These protocols require updating for multidetector CT. Such protocols should be reviewed on a regular basis. It would be preferable for criteria such as RECIST to provide a guideline for the selection of imaging modalities and technologies and standard mechanisms for creating disease- and modality-specific imaging protocols. In this manner, the precise clinical trial protocols could be defined based upon these guidelines and the more general RECIST criteria would still be relevant. For example, as outlined in RECIST, the specific concept of slice thickness relative to spiral CT data acquisition is already obsolete, but a general mechanism of defining slice thickness relative to lesion size would still be relevant.
  - In the RECIST criteria, the authors state that it is not acceptable to switch between windows when measuring lesions, but ‘in the lung it does not really matter whether lung or soft tissue windows are used for intraparenchymal lesions’. We do not agree with this statement, which may be misleading to the uninitiated. It is possible to see substantial differences in size and texture of lung lesions by viewing them on lung and soft tissue windows. It is our recommendation that the window settings must be optimised to best visualise the lesion, and lesions should be viewed on multiple window settings but consistent windowing must be used for subsequent assessments. We believe that the lung window settings are the optimal ones for obtaining measurements of all parenchymal lesions.
  - There are new, novel methods of assessing disease response in areas, such as bone, that are difficult to assess, and have been considered nonmeasureable according to RECIST. For instance, there is now evidence that MR imaging may be a useful method of assessing response to therapy both in breast cancer and in other tumours (Carlson *et al*, 1995; Brown *et al*, 1998; Ciray *et al*, 2001). Other tumour types including prostate are not amenable to assessment by RECIST since the burden of disease in many patients is in the bone.
  - Although the sum of all lesions measured provides an indication of total tumour burden, such arithmetic sums may disguise differential tumour response. Such discrepancy of response has not been addressed with the new RECIST criteria.
  - A guideline concerning the number of lesions to include in measurement is helpful and an advantage of RECIST over the WHO criteria, which provided no directives in this regard. In fact, cooperative groups have adopted a specific number of lesions by appending the RECIST criteria. However, to our knowledge, there is no evidence provided for the scientific basis of the need to measure multiple lesions (up to 10 lesions). Interestingly, Cheson in a report of an international workshop to standardise response criteria for non-Hodgkin's lymphomas noted a paucity of data for the number of lesions to measure and included an arbitrary number in their criteria, but left open to further discussion. The critical issue is in fact the number of lesions that are needed to be measured to have a consistent response assessment—either by the same observer or another observer, that is, to decrease variability in response assessment based upon choosing a different set of lesions. Preliminary data and experience would suggest that for certain tumours it is not necessary to measure as many as 10 lesions (Schwartz *et al*, 2003b).

- We agree that intravenous administration of contrast medium is an essential component of examination of the liver. However, there are several potential pitfalls in measurements of liver lesions. For example, there is no indication of whether liver metastases should be measured including the ‘enhancing rim’ or whether only the ‘central lower density component’ should be measured on CT.
- It is difficult, but becoming increasingly important, to standardise the approach to administering intravenous contrast medium between different centres. We appreciate that this would be difficult, but working towards a standardised approach is of considerable importance. For example, measurement of a liver lesion in the ‘arterial phase’ of enhancement will produce a different measurement from that produced if the lesion is measured in the ‘portal phase’ of enhancement (Figure 3

**Figure 3:**
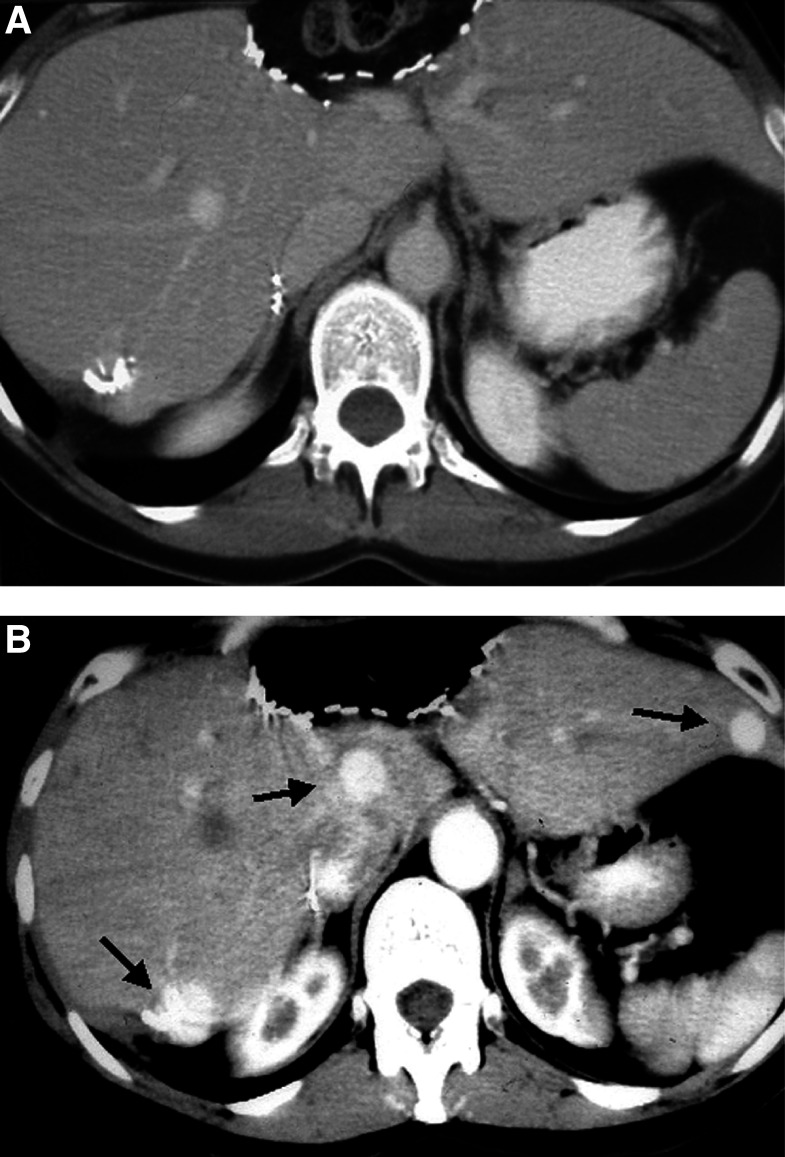
Visualisation of liver metastases is greatly influenced by phase of contrast administration. Note nonvisualisation of lesions in the equilibrium phase (**A**), which are seen in the arterial phase (**B**) (arrows).

). Although the authors state that ‘a suitable contrast agent should be given so that the metastases are demonstrated to best effect and a consistent method is used on subsequent examinations for any given patient’, there is no attempt to standardise such an approach across a clinical trial. Therefore, in one institution, a completely different method of assessing liver metastases could be conducted compared with another institution within the same study, leading to major discrepancies and probable error! (Nazarian *et al*, 1999).
- It is important to raise our concern regarding the frequency and intensity of imaging required to assess therapeutic response as it relates to radiation exposure. In many clinical protocols, sequential examinations of the chest, abdomen and pelvis are required following every two cycles of chemotherapy. The adoption of multidetector CT scanning and thin collimation slices raises the important issue of radiation dosage to the patient, a topic that is becoming increasingly important, and addressed by other consensus statements in the imaging community (Brix *et al*, 2003; Mayo *et al*, 2003).
- There is room for debate and discussion regarding the need for CT scans of the chest, abdomen, pelvis and sometimes the neck during follow-up in patients with certain tumours in whom multiple CT scans are performed over a considerable length of time (e.g. lymphoma or testicular tumours). Reduction in the radiation dose could be achieved by limiting follow-up CT scans to the area(s) of disease, or the most likely sites of relapse (Thomas *et al*, 1982; Wright and White, 1999; Boger-Megiddo *et al*, 2002). Radiation dose is also of great concern in the follow-up of paediatric tumours.
- Unfortunately, many clinical trials are discussed and agreed with clinical principal investigators and diagnostic radiologists are only involved after the protocol has been agreed. It is the view of the ICIS that a radiologist should be a prominent member of the clinical research team when new clinical trials are being developed. It should be the responsibility of both the principal investigator and of industry to recognise the pivotal role of diagnostic imaging, not only in evaluation of response but also in setting up clinical trials from the outset.
- The ICIS would suggest that in selected trials, off-site independent review of imaging data is undertaken by expert oncological radiologists who have experience in this subspecialty of diagnostic radiology. This is frequently not the case. The RECIST criteria have been set up in order to improve the accuracy and reproducibility of reporting of clinical trials among multiple sites and trials, but if the adoption of such criteria is unworkable for technical and practical purposes, the major objective is unlikely to be realised. Currently, commercial organisations, contract research organisations, cooperative groups and governmental agencies conduct independent response assessment. Many aspects of these external reviews, including, but not limited to, the use of film versus digital image data, number of external reviewers, format of the external review, use of clinical data in addition to image data, are not standardised. This lack of uniformity may lead to differences in response assessment, independent of the measurement methodology utilised. These differences have not been well studied or even documented in the oncology or radiology literature. Observer variability is also a major issue in response assessment. Data suggest that measurements made by a limited number of experienced observers should increase the accuracy in quantitative analysis of medical images (Belton *et al*, 2003).
- It is interesting to note that several radiologists working in cancer imaging have stated that recent trials have been set up with protocols that state that either RECIST or WHO or modifications of both criteria may be used. Further, disease-specific guidelines have been proposed, which are being used in many cooperative group trials (Cheson *et al*, 1999). It seems that a standardised approach is far from being realised.

We propose that a working party of the ICIS enters a dialogue with the authors of the New Guidelines to Evaluate Response of Solid Tumours (RECIST) in order to modify and update the guidelines in accordance with current opinion as outlined above. We propose that such dialogue should be ongoing so that new information from existing and new technology can be introduced into the guidelines in the future in a timely and appropriate manner. For example, there is clearly a growing need to introduce a multimodality approach to monitoring tumour response, thereby integrating morphological and functional tumour measurements. The rapidly growing application of positron emission tomography (PET), PET/CT and MRI to monitoring tumour response provides this opportunity and opens the way to a new and exciting era in cancer imaging.
